# Content Validity of the Modified Functional Scale for the Assessment and Rating of Ataxia (f-SARA) Instrument in Spinocerebellar Ataxia

**DOI:** 10.1007/s12311-024-01700-2

**Published:** 2024-05-07

**Authors:** Michele Potashman, Katja Rudell, Ivanna Pavisic, Naomi Suminski, Rinchen Doma, Maggie Heinrich, Linda Abetz-Webb, Melissa Wolfe Beiner, Sheng-Han Kuo, Liana S. Rosenthal, Theresa Zesiwicz, Terry D. Fife, Bart P. van de Warrenburg, Giovanni Ristori, Matthis Synofzik, Susan Perlman, Jeremy D. Schmahmann, Gilbert L’Italien

**Affiliations:** 1 Biohaven Pharmaceuticals, Inc, New Haven, CT USA; 2 COA Science, Parexel International, London, UK; 3 Patient-Centered Outcomes Assessments, Macclesfield, UK; 4 Department of Neurology, Columbia University, New York, NY USA; 5 Department of Neurology, Johns Hopkins School of Medicine, Baltimore, MD USA; 6 Department of Neurology, Ataxia Research Center, University of South Florida, Tampa, FL USA; 7 Department of Neurology, Barrow Neurological Institute, University of Arizona College of Medicine, Phoenix, AZ USA; 8 Department of Neurology, Radboud University Medical Centre, Nijmegen, The Netherlands; 9 Department of Neurosciences, Mental Health and Sensory Organs, Sapienza University of Rome, Rome, Italy; 10 Division of Translational Genomics of Neurodegenerative Diseases, Hertie Institute for Clinical Brain Research and Center of Neurology, University of Tübingen, Tübingen, Germany; 11 Department of Neurology, David Geffen School of Medicine at UCLA, Los Angeles, CA USA; 12 Ataxia Center, Laboratory for Neuroanatomy and Cerebellar Neurobiology, Department of Neurology, Massachusetts General Hospital, Boston, MA USA

**Keywords:** Spinocerebellar ataxia, f-SARA, Clinical outcome assessment, Concept elicitation, Cognitive debriefing

## Abstract

The functional Scale for the Assessment and Rating of Ataxia (f-SARA) assesses Gait, Stance, Sitting, and Speech. It was developed as a potentially clinically meaningful measure of spinocerebellar ataxia (SCA) progression for clinical trial use. Here, we evaluated content validity of the f-SARA. Qualitative interviews were conducted among individuals with SCA1 (*n* = 1) and SCA3 (*n* = 6) and healthcare professionals (HCPs) with SCA expertise (USA, *n* = 5; Europe, *n* = 3). Interviews evaluated symptoms and signs of SCA and relevance of f-SARA concepts for SCA. HCP cognitive debriefing was conducted. Interviews were recorded, transcribed, coded, and analyzed by ATLAS.TI software. Individuals with SCA1 and 3 reported 85 symptoms, signs, and impacts of SCA. All indicated difficulties with walking, stance, balance, speech, fatigue, emotions, and work. All individuals with SCA1 and 3 considered Gait, Stance, and Speech relevant f-SARA concepts; 3 considered Sitting relevant (42.9%). All HCPs considered Gait and Speech relevant; 5 (62.5%) indicated Stance was relevant. Sitting was considered a late-stage disease indicator. Most HCPs suggested inclusion of appendicular items would enhance clinical relevance. Cognitive debriefing supported clarity and comprehension of f-SARA. Maintaining current abilities on f-SARA items for 1 year was considered meaningful for most individuals with SCA1 and 3. All HCPs considered meaningful changes as stability in f-SARA score over 1–2 years, 1–2-point change in total f-SARA score, and deviation from natural history. These results support content validity of f-SARA for assessing SCA disease progression in clinical trials.

**Supplementary Information:**

The online version contains supplementary material available at 10.1007/s12311-024-01700-2.

## Introduction

Spinocerebellar ataxias (SCAs) are a dominantly inherited heterogeneous group of rare disorders that cause progressive neurodegeneration of the cerebellum and spinal cord [1–3]. Almost 50 different SCA genotypes have been identified, each with a distinct pathophysiology and clinical profile. Of these, SCA types 1, 2, 3, and 6 have been considered the most common worldwide [1, 3–7]. The recent identification and characterization of the newly discovered SCA27b variant has suggested that this may account for a substantial proportion of previously unexplained late-onset dominant and sporadic cerebellar ataxias, though its global prevalence remains to be established [4, 5, 8, 9]. Whereas symptom manifestation and disease trajectory vary across SCA types, all share the cardinal features of cerebellar dysfunction, which includes progressive lack of voluntary motor coordination, gait impairment, loss of balance and associated falls, and speech and swallowing difficulties [1, 10–12]. In addition to affecting physical functioning, symptoms impair independent ability to conduct activities of daily living (ADLs), which increases reliance on caregivers and severely impacts patient quality of life [13–16]. Patient life expectancy varies widely between SCA types [7, 17].

To reliably measure the severity and progression of cerebellar ataxia, notably SCA, the Scale for the Assessment and Rating of Ataxia (SARA) was developed by a panel of expert clinicians to provide semi-quantitative scoring of patient gross and fine motor function [18]. The SARA evaluates 8 items concerning gait, stance, sitting, speech, and upper and lower limb coordination. It provides a combined total score that indicates disease severity, with higher scores denoting more severe disease. A number of patient registries and clinical studies have used the SARA as an outcome measure to date to assess the impact of pharmacologic and/or non-pharmacologic therapies on SCA symptom progression [19–32]. Recently, the SARA was modified to serve as a primary endpoint in a randomized clinical trial of individuals with SCA [33]. Accounting for feedback from discussions with the Food and Drug Administration (FDA), and upon analysis of US natural history data from the Clinical Research Consortium for the Study of Cerebellar Ataxia and a phase 2 study of troriluzole [34], removal of the 4 appendicular items from the original SARA was implemented. These items were not considered sensitive for measurement of meaningful change in a clinical trial setting conducted over 48 weeks.

The resulting modification of the SARA, the functional SARA (f-SARA), is a 4-item scale that assesses Gait, Stance, Sitting, and Speech. Each of the 4 items is rated on an ordinal scale from 0 to 4, where 0 indicates normal or unimpaired function, and higher responses indicate progressive impairment. The total f-SARA score is the sum of the 4 individual items (16 points).

When developing or adapting a clinical outcome assessment (COA), it is important to collect patient perspectives on their lived experience of the disease of interest, as well as clinical perspectives on the temporal progression of the disease, to support the content validity of the measure [35, 36]. In addition, cognitive debriefing and discussions centered around what constitutes meaningful changes in the context of the disease are important steps in establishing the content validity of a new or modified COA. These discussions ensure that the measure has the potential to assess meaningful changes in patient-experienced symptoms reliably [35, 36].

The f-SARA was developed to support the primary endpoint in a phase 3 study evaluating the efficacy of troriluzole on ataxia symptoms in individuals with SCA (NCT03701399; trial registration date: October 8, 2018), but the content validity of the items that comprise the f-SARA (i.e., comprehensiveness, relevance, comprehension, and understandability) remains to be determined. To address this need, we conducted qualitative interviews with individuals with SCA and healthcare professionals (HCPs) with expertise in treating SCA to assess the content validity of the f-SARA and to explore what constitutes clinically meaningful changes in SCA symptoms.

## Patients and Methods

### Study Design

Qualitative interviews were conducted with individuals diagnosed with SCA in the United States and with HCPs who treat SCA in the United States and Europe. Interviews were designed according to the FDA Patient Focused Drug Development Guidance [37–39] to assess individuals with SCA and HCPs’ perspectives of the f-SARA in terms of comprehensiveness, content validity, item relevance, and ability to measure meaningful changes. Interviews consisted of concept elicitation and cognitive debriefing phases.

### Participants

HCPs who met the eligibility criteria and were considered experts in assessing and treating individuals with SCA were identified (eligibility criteria shown in Supplementary Table 1). There were 2 cohorts of HCPs included in the interviews. The first from the United States with prior experience of using the f-SARA instrument through participation in the phase 3 study evaluating troriluzole efficacy (NCT03701399) (f-SARA previously exposed; HCPs 1–5), and the second from Europe with no prior clinical experience with the f-SARA instrument (f-SARA newly exposed; HCPs 6–8). All HCPs had extensive experience with COAs in SCA including the SARA.

Eligible individuals aged 18–75 years with any SCA type were recruited via clinician referral or self-referral from a patient advocacy organization (National Ataxia Foundation) (participant eligibility criteria shown in Supplementary Table 2).

### Interview Process

Discussion guides were developed for semi-structured interviews with individuals with SCA and HCPs, respectively (Supplementary Table 3). Interview questions were focused primarily on symptoms and impacts associated with SCA. Interviews were conducted in English via video call and were semi-structured lasting approximately 120–180 min over either 1 or 2 sessions. Interviews also included discussions assessing 2 other SCA COAs, which will be reported elsewhere.

**Fig. 1:**
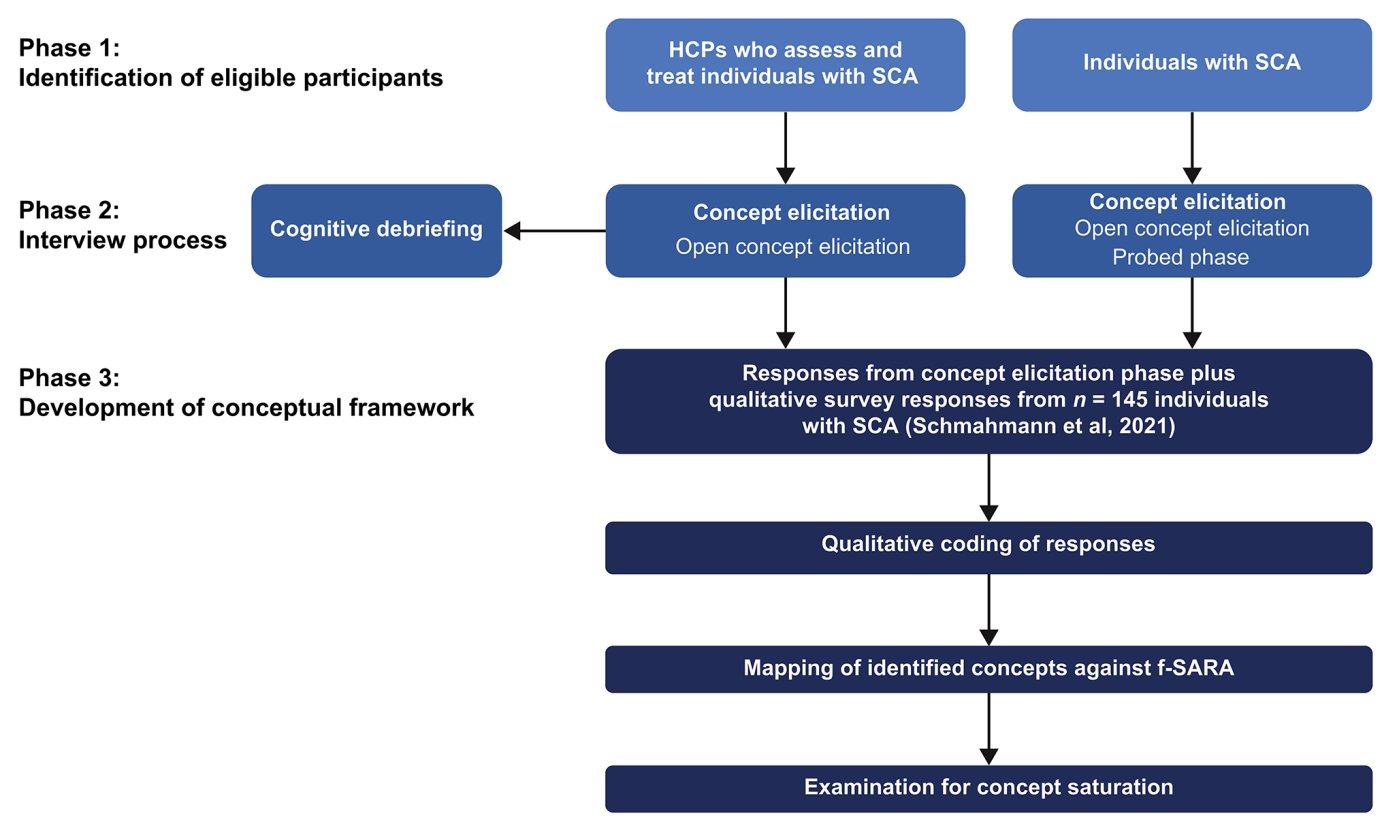
Study design and development of the conceptual framework Abbreviations: f-SARA modified functional Scale for the Assessment and Rating of Ataxia, HCP healthcare professional, SCA spinocerebellar ataxia

During the interview, demographic and health information was initially collected from individuals with SCA, and pertinent demographic information was ascertained for the HCPs. Participants then underwent a concept elicitation session to explore the lived and observed experiences, and daily functioning abilities of individuals with SCA. The open concept elicitation phase was followed by a set of probes designed to query the symptoms HCPs regarded as most common. Probed concepts were identified from Schmahmann et al. [40], Potashman et al. [41], and clinician input. Relevant concepts were then inserted into the interview discussion guide as probes to capture the patient experience of SCA (Fig. 1). A sample of the interview guide is presented in Supplementary Table 3. Based on data from Schmahmann et al. [40] and Potashman et al. [41], survey responses from 145 individuals with SCA were qualitatively coded using ATLAS.Ti v22 software. This codebook was then used to analyze the frequency and relevance of concepts identified in the semi-structured interviews. Examination for saturation was assessed at the time of semi-structured interview data analysis.

Finally, cognitive debriefing was employed to evaluate the understandability, relevance, and comprehensiveness of the f-SARA in relation to SCA. Relevance and comprehensiveness of the f-SARA was assessed by mapping concepts discussed by HCPs and individuals with SCA against the items included in the f-SARA to identify factors that were considered important from a disease severity perspective. Meaningful changes as measured through the lens of the instrument, as well as the relevance of specific symptoms and impacts in the context of SCA, were also explored in the interviews.

### Sample Size Calculation

In clinical outcomes research studies, a general concept elicitation and cognitive debriefing study is conducted until saturation is reached. Saturation analyses are performed to confirm that there are no further additional concepts identified. Typically, saturation may be reached within 10–15 interviews [42]. The low prevalence of rare diseases often only allows for small patient sample sizes. Consequently, the data generated from this study were a supplement to previous qualitative work carried out by Schmahmann et al. [40] and Potashman et al. [41]. The data generated here, in combination with the results from the open-ended survey [40, 41], may be considered acceptable to verify the validity and relevance of the f-SARA in a small (< 10 patients) sample of patients with SCA.

### Data Analysis

A descriptive content analysis was used to analyze interviews with HCPs and individuals with SCA, specifically to identify themes or concepts that were elicited from the interviews after they were transcribed. Interviews were conducted via a web-based platform, audio recorded and then transcribed. Transcripts were anonymized and coded using ATLAS.Ti v22 software. A codebook and qualitative analysis plan were developed and used to code each transcript. Briefly, coding dictionaries were developed, using an iterative process after completion of approximately 3 interviews. One codebook was used for analyzing HCP interview transcripts and one for analyzing the interview transcripts from individuals with SCA. The coding process was guided by established qualitative research methods [43]. Multiple coders reviewed the transcripts to minimize bias. Cognitive debriefing analyses were conducted in accordance with standard procedures to evaluate participants’ understanding of the measures [44].

### Ethical Considerations

The study (BHV-4157-SCA-VAL) was approved by a centralized independent Institutional Review Board (IRB; Salus Institutional Review Board, Austin, TX, USA). IRB approval was not required for HCP interviews conducted in the United States or Europe. All eligible individuals with SCA and the HCPs provided informed consent to participate in the interviews and could withdraw at any time. HCPs received consultancy fees for participating in the interviews.

## Results

### Participant Demographics and Characteristics

#### HCPs

Eight HCPs (all neurologists) from the United States (f-SARA previously exposed; *n* = 5, corresponding to HCPs 1–5) and Europe (f-SARA newly exposed; *n* = 3, corresponding to HCPs 6–8) with expertise in SCA were recruited from centers of excellence in treating ataxias and related cerebellar disorders. Interviews with US HCPs were conducted between July and September 2022, and those with European HCPs were conducted between June and August 2023. Of 8 participating HCPs, most were male (*n* = 5; 62.5%) (Table 1). The number of individuals with all genotypes of SCA that HCPs reported they had treated in clinical practice over the course of their career ranged from “80” to “thousands.”

**Table 1:** Summary of HCP characteristics

Demographics	f-SARA previously exposed (USA) (*n* = 5)	f-SARA newly exposed (Europe) (*n* = 3)	Total (*N* = 8)
Sex, *n* (%)			
Male	2 (40.0)	3 (100.0)	5 (62.5)
Female	3 (60.0)		3 (37.5)
Specialty/profession, *n* (%)			
Neurology	2 (40.0)	3 (100.0)	5 (62.5)
Neurology and movement disorders	3 (60.0)		3 (37.5)
Approximate number of individuals treated with SCA over career course, *n* (%)			
50–100 individuals	2 (40.0)	1 (33.3)	3 (37.5)
101–200 individuals	1 (20.0)		1 (12.5)
201–500 individuals	1 (20.0)		1 (12.5)
“Hundreds”	1 (20.0)	1 (33.3)	2 (25.0)
“Thousands”		1 (33.3)	1 (12.5)

Abbreviations: f-SARA modified functional Scale for the Assessment and Rating of Ataxia, HCP healthcare professional, SCA spinocerebellar ataxia

#### Individuals with SCA

Overall, 7 individuals with SCA from the United States participated in the interviews (SCA1, *n* = 1; SCA3 *n* = 6), which were conducted between October and December 2022. Most participants were male (*n* = 4; 57.1%) (Table 2), and all (100%) had genetically confirmed SCA diagnoses.

**Table 2:** Summary of demographics and clinical characteristics for individuals with SCA1 and 3

Demographics	Total (*N* = 7)
Age (years)	
Mean (range)	51 (34–65)
Sex, *n* (%)	
Male	4 (57.1)
Female	3 (42.9)
Ethnicity, *n* (%)	
Asian	2 (28.6)
White	4 (57.1)
Black or African American	1 (14.3)
Educational level, *n* (%)	
High school	2 (28.6)
Bachelor’s degree	2 (28.6)
Graduate degree	3 (42.9)
Work status, *n* (%)	
Working full-time	5 (71.4)
Retired	1 (14.3)
Disability benefit	1 (14.3)

Clinical characteristics	
SCA diagnosis, *n* (%)	
SCA3	6 (85.7)
SCA1	1 (14.3)
Age at diagnosis in years	
Mean (range)	44 (31–56)
Diagnosis type (*not mutually exclusive*), *n* (%)	
Genetic testing	7 (100.0)
Family history^a^	6 (85.7)
Clinical/medical/other diagnosis^a^	5 (71.4)
Severity of SCA, *n* (%)	
Stage 0 (no gait/walking difficulties)	1 (14.3)
Stage 1 (gait difficulties but can walk)	0 (0)
Between Stage 1 (gait difficulties but can walk) and Stage 2 (cannot walk without permanent use of a walking aid/help)	4 (57.1)
Stage 2 (cannot walk without permanent use of a walking aid/help)	1 (14.3)
Stage 3 (in a wheelchair)	1 (14.3)

^a^All individuals with a family history and/or a clinical/medical/other diagnosis also indicated genetic testing diagnosis

Abbreviation: SCA spinocerebellar ataxia

### SCA Symptoms, Progression, and Impact on Daily Function

#### Perspectives from HCPs

To ascertain how SCA progresses and the most important symptoms impacting the function of individuals with SCA, HCPs were asked to describe the symptoms of SCA; how they would define mild, moderate, and severe stages of disease; and what they would consider the most concerning limitation in each of these stages. Overall, SCA was described as a multi-domain progressive disease impacting motor function, speech, vision, and cognition, which affects every aspect of daily life (Supplementary Table 4).

HCP5 described the impact of SCA on individuals’ daily lives: *“It affects every aspect of their daily lives. It affects their ability to communicate. It affects their ability to ambulate and to walk. It affects fine finger coordination, like fine motor skills. So, that’s definitely a problem for them. Also, people with spinocerebellar ataxia have problems, often with cognitive issues.”*

HCPs defined disease severity as an increasing presence of symptoms, which impacts individuals’ autonomy and ability to live independently. Mild disease was characterized by few symptoms and little to no impact on ADLs. Individuals with severe disease were considered to be extremely impaired and limited in ADLs (e.g., needing to use a wheelchair and requiring assistance with most or all activities). Three HCPs (37.5%) characterized disease severity by the use of a walker or wheelchair for mobility. Additional symptoms such as speech, vision, and balance were reported by 4 HCPs (50.0%) as factors that characterize disease severity.

When asked to provide the 5 most impactful symptoms/issues affecting the daily life of individuals with SCA, HCPs spontaneously reported the following concepts: difficulty with walking, speech, fine motor accuracy, and balance, and social/work impact (Table 3). Spontaneously reported concepts included 3 of the 4 f-SARA items: Gait, Stance, and Speech.

**Table 3:** Top 5 reported concepts by HCPs that impact daily functioning in individuals with SCA

Concept	Total number of HCPs, *n* (%)
Walking	8 (100.0)
Speech	8 (100.0)
Fine motor accuracy	5 (62.5)
Balance	5 (62.5)
Social/work impact	5 (62.5)

Abbreviations: HCP healthcare professional, SCA spinocerebellar ataxia

HCP6 described the top 5 SCA symptoms that impact individuals’ daily functioning: “*Walking, in particular walking stairs, walking on uneven underground surfaces; standing without swaying, standing stable, that’s number 2. Number 3 is writing, and closing, and using a key. That’s impairment number 3. And number 4 is swallowing deficits, coughing, and choking when drinking or eating. And number 5 is speaking unclearly with a slurred voice and having to repeat statements.”*

#### Perspectives from Individuals with SCA

To establish the most important SCA disease-related experiences from the perspective of individuals with SCA1 and 3, individuals were asked to report their signs, symptoms, and impacts on daily life. Probing questions were then asked regarding a set of specific concepts recommended by HCPs as prominent, if they were not spontaneously mentioned. A total of 85 sign, symptom, and impact concepts were reported during the interviews (*n* = 66 spontaneously reported concepts by ≥ 1 individual, and *n* = 18 concepts confirmed with probes) (Supplementary Table 5). All individuals with SCA spontaneously reported difficulties with walking and balance. Other signs/symptoms/ADL impacts frequently spontaneously reported (≥ 50.0% of individuals) were falls (*n* = 6/7), tired/fatigued (*n* = 5/7), difficulty working (*n* = 5/7), challenges with social life (*n* = 5/7), difficulty being understood (*n* = 4/7), emotional dysfunction (*n* = 4/7), difficulty driving (*n* = 4/7), and vision impairments (*n* = 4/7). When adding in the probed items, the additional concepts of difficulties dressing (*n* = 5/7), difficulties swallowing/choking (*n* = 5/7), difficulties climbing stairs (*n* = 5/7), difficulties exercising (*n* = 4/7), difficulties with housework (*n* = 4/7), unable to do usual activities (*n* = 4/7), difficulties sitting for long periods (*n* = 4/7), issues with bladder function (*n* = 4/7), and requiring assistance to use the toilet (*n* = 4/7) were reported by ≥ 50.0% of individuals.

In addition, over the course of the interviews, all 7 individuals with SCA shared feelings of anxiety, fear of falling, difficulty dealing with the condition alone, nervousness during work calls, trauma from falls, embarrassment during coughing spells, laziness, and not having initiative.

Individual 7 commented regarding important and meaningful issues related to SCA: *“And that’s what really bothers me day in and day out. Thinking that I may not have anybody to take care of me. And I won’t be able to even speak to communicate.”*

Individual 3 commented regarding important and meaningful issues related to SCA: *“You give out the perception that you’re drunk a lot, which causes people to treat you differently, and causes people to look at you differently.”*

**Fig. 2:**
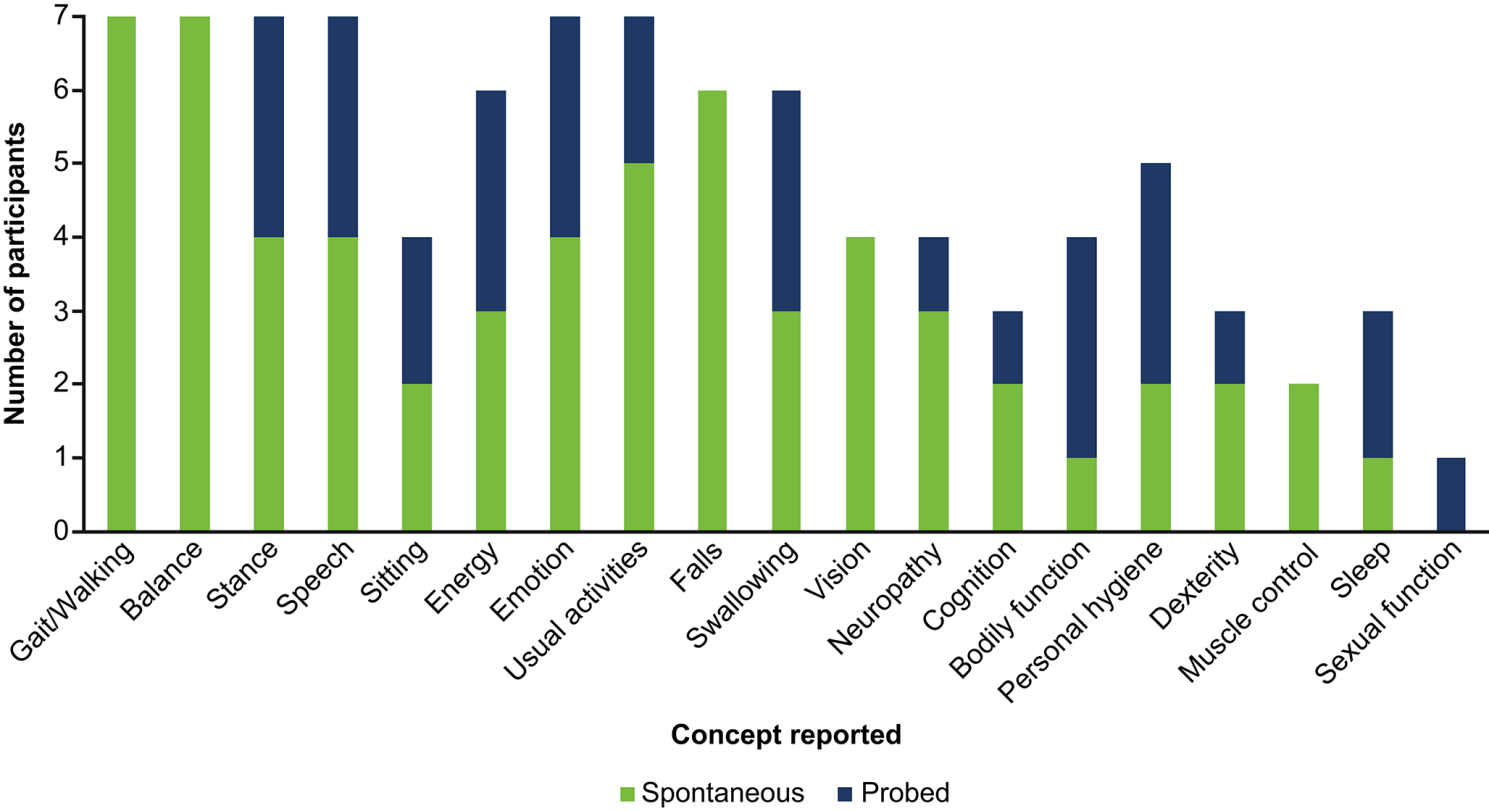
Overarching concepts identified in interviews with individuals with SCA Abbreviation: SCA spinocerebellar ataxia

It was not possible to determine whether saturation was achieved for the entire sample due to the small sample size. However, at least 1 spontaneously reported item from each f-SARA concept was elicited during the first three-quarters of the interviews (i.e., prior to interview of Individual 6). Additionally, on consideration of both the spontaneously reported and probed items, several signs, symptoms, and impacts were reported by all 7 individuals with SCA including difficulties with walking (including abnormal gait), stance, balance, speech (e.g., slurred speech and speech production difficulties), and working; feeling tired or fatigued; and emotional dysfunction (Fig. 2).

Following this, individuals with SCA were asked to report the symptoms they considered to be most bothersome and what impact they believed these symptoms had on their daily lives (Supplementary Table 6). The most bothersome symptoms were neuropathy (*n* = 4/7) and gait and/or balance (*n* = 3/7). Other symptoms reported as most bothersome included vision (*n* = 2) and communication/speech problems (*n* = 2). Impacts to work life (*n* = 1), social life (*n* = 1), and sleep (*n* = 1), and the prospect of a neurological decline in the future (*n* = 1) were also reported as meaningful.

Individual 5 commented regarding the most bothersome SCA symptoms: *“Well, the neuropathy is the most bothersome because I cannot sleep.”*

Individuals with SCA were then asked probing questions on the detailed aspects of the specific symptom domains that most affected their lives and were most bothersome (Supplementary Table 7). Among those who experienced gait and walking difficulties, “general difficulties walking” was considered the most bothersome (*n* = 5/7) and particularly important (*n* = 4/7) symptom. A proportion of individuals reported the “sometimes requiring a walking aid” as the most bothersome (*n* = 2/3) and important (*n* = 3/3) symptom. Three of the 6 individuals included “trouble keeping balance” as the most bothersome (*n* = 3/6), with 2 indicating it as the most important (*n* = 2/7) symptom. Of those individuals experiencing issues with sitting, most indicated general difficulties with sitting to be the most bothersome (*n* = 3/4) symptom, and half reported this to be of particular importance (*n* = 2/4). Two individuals reported “requiring back support to sit,” though neither indicated this to be the most bothersome symptom; 1 individual (*n* = 1/2) reported this to be the most important. For speech, more than half of the individuals with SCA reported that their speech being “occasionally difficult to understand” was the most bothersome (*n* = 4/7) and most important symptom (*n* = 4/7). Of those reporting difficulties with swallowing, most indicated that choking was the most bothersome (*n* = 3/5) symptom, and 2 considered this to be the most important (*n* = 2/5). For energy, 2 individuals considered fatigue as the most bothersome symptom (*n* = 2/7); most reported that fatigue was particularly important (*n* = 5/7).

### Relevance of f-SARA Concepts

#### Perspectives from HCPs

All HCPs (*n* = 8) spontaneously described the f-SARA concepts of Gait, Stance, and Speech items as relevant for tracking disease progression, and included these concepts in overall disease staging of mild, moderate, or severe SCA. Furthermore, they confirmed that these items reflected meaningful aspects of the lives of individuals with SCA. Sitting, the fourth f-SARA item, was not spontaneously mentioned by any of the HCPs and responses regarding the relevance of Sitting were varied when HCPs were asked probing questions. In addition, HCPs suggested a few concepts to include that could improve the clinical relevance of the f-SARA, namely fine motor accuracy/dexterity (*n* = 6; 75.0%), vision problems (*n* = 4; 50.0%), and swallowing (*n* = 3; 37.5%). HCPs further suggested that inclusion of relevant items evaluating the impact of symptoms on ADLs would promote a more detailed assessment of disease severity.

HCP5 on their overall impression of the f-SARA: *“Well, the f-SARA is a good, quick tool to evaluate ataxia symptoms and neurological function in a spinocerebellar ataxia patient.”*

HCP6 on their overall impression of the f-SARA: *“So, what I do like is that the 4 domains which are there, they are indeed of key importance for ataxia: gait, stance, sitting, speech. Those are 4 domains, not only from [a] neurological perspective, but indeed from a patient’s daily life perspective. Those are 4 domains of high impact for the patient. So, that’s a positive point.”*

HCP4 on their overall impression of the f-SARA: *“I think that [fine motor dexterity] would be a better addition to the f-SARA than Sitting. Because in my observation, very few people had substantial sway when they’re sitting.”*

#### Perspectives from Individuals with SCA

Three of the 4 concepts in the f-SARA, Gait, Stance, and Speech, were considered relevant by all 7 individuals with SCA. Sitting was reported as meaningful by 3 individuals. When asked to rank the relevance of concepts covered by the f-SARA using a 5-point rating scale (from 0 = “not at all” relevant to 4 = “extremely” relevant), 6 of 7 individuals reported that most items were relevant (each concept ≥ 1 [a little relevant]) in the context of their experience of SCA. Individuals with SCA ranked difficulties with gait and walking (*n* = 7) as extremely relevant (median score of 4), followed by difficulty with standing/balance (*n* = 6; median score of 3); the median score for both speech (*n* = 6) and sitting (*n* = 7) was 2. In addition to rating the relevance of f-SARA concepts, individuals with SCA were asked to rank the 3 most important concepts from the f-SARA. Most individuals reported Gait (*n* = 6) as the most important, followed by Stance (*n* = 3), Speech (*n* = 2), and Sitting (*n* = 1). Those who considered Gait to be the most important concept generally reported that difficulties with mobility impacted their sense of independence. Individuals who reported balance as the most important concept indicated frequently falling affected their ability to enjoy hobbies. For those who regarded difficulties with Speech as the important concept, it was highlighted that progressively losing the ability to communicate with others would negatively impact quality of life. The individual who reported Sitting as the most important noted that it was currently difficult to sit because of the pain.

Individual 6 commented on the most important f-SARA concept: *“I would say the difficulty walking and the gait would be most important. Gait and walking around is essential to seeing the world and being part of the world around you. And if you can’t move and you can’t walk, you can’t be out in the world.”*

Individual 3 commented on the most important f-SARA concept: *“Difficulties with gait and walking is definitely number 1. It’s like effort every single step you take not to fall, which is frustrating because your knees buckle.”*

### Capturing Meaningful Change and Stability Using the f-SARA: Perspectives from HCPs

#### Meaningful Change in f-SARA Scores

To assess whether the f-SARA captures changes perceived as clinically meaningful, HCPs were asked to describe what changes in the f-SARA score would be most meaningful when prescribing a therapy to individuals with SCA.

Considering meaningful improvement of f-SARA items, all HCPs (*n* = 8/8) reported that a 1-point improvement in Gait would reflect a meaningful change (Supplementary Table 9). For Stance, the majority of HCPs (*n* = 6/8) reported that a 1-point improvement would be meaningful. The additional HCPs considered meaningful change to be a 2-point improvement (*n* = 1) or reducing the need for aids/supports (*n* = 1). Most HCPs (*n* = 6/8) considered a 1-point improvement on the Speech item as meaningful; however, 1 HCP clarified their response by stating that a 1-point change on Speech would be a meaningful improvement when the item score changed from 3 to 2 or 1. The 2 additional HCPs considered that meaningful improvement on the Speech item would be a 1- to 2-point change. Of interest, 1 HCP indicated that a 1-point improvement on any of the Gait, Stance, or Sitting items would be of particular importance. Defining meaningful change for the Sitting item was considered challenging by the majority of HCPs (*n* = 6/8) because the ability to sit does not typically deteriorate linearly, and an individual’s ability to sit may vary day to day.

HCP8 commented on meaningful improvement in f-SARA items: *“If you are able to change from in need of support to going down to no need of support, that is a relevant change.”*

HCP5 commented on meaningful improvement in f-SARA items: *“I would say a 1-point improvement on the Gait would be the most meaningful.”*

For meaningful worsening on the Gait item, most HCPs (*n* = 7/8) considered a 1-point decline to be meaningful; however, 1 HCP reported that it was difficult to quantify. Among the Stance, Sitting, and Speech items, all HCPs previously exposed to the f-SARA (*n* = 5/5) considered a 1-point decline to be meaningful. Of note, 4 HCPs previously exposed to the f-SARA (*n* = 4/5) spontaneously reported that the 0- to 4-point scoring scale of the f-SARA may be unlikely to detect small (< 1-point) changes that would be meaningful to individuals with SCA. HCPs newly exposed to f-SARA did not quantify meaningful decline on the Stance, Sitting, and Speech items, stating that it was anchored to f-SARA natural history changes that they did not have experience with yet.

HCP6 commented on meaningful worsening in f-SARA items: *“In the Gait item, a 1-point change is a huge thing.”*

HCP1 commented on meaningful changes in f-SARA items: *“Well, I think a change certainly for Gait, Stance, and Sitting, a full point for any of these areas would obviously be clinically meaningful. So, I think the scoring levels definitely reflect clinically meaningful changes in an exam that would be hard to overlook.”*

For the total f-SARA score, all 8 HCPs regarded minimum meaningful improvements or worsening as a 1- to 2-point change; however, intra-individual meaningful changes on total f-SARA score differed between previously-exposed and newly-exposed HCPs. Previously-exposed HCPs considered meaningful improvement or worsening as a 1- to 2-point change in total f-SARA score, whereas newly-exposed HCPs reported that meaningful change would be anchored to natural history changes. Most HCPs (*n* = 5/8) specified that a minimum of a 1-point change in the f-SARA total score would be regarded as meaningful improvement; however, responses ranged from 1 to 4 points. One HCP noted that a worsening of the f-SARA total score by 1 to 2 points was aligned with the natural history of disease progression over 1 year. Of the HCPs who indicated that a 2-point change in f-SARA total score would be meaningful (*n* = 3/8), 1 qualified their statement by reporting that a 1-point change may not represent a real effect, and another stated that a 1-point change on > 1 domain would be meaningful. Additionally, 1 HCP indicated that meaningful improvements in the f-SARA total score may be relative to the baseline of each individual (e.g., worsening of 1 point by going from 13 to 14 may not be meaningful, but an improvement from 6 to 5 points may be meaningful). A minimum of a 1-point worsening in total f-SARA score was reported by most HCPs (*n* = 6/8) as meaningful, with responses ranging from 1 to 4 points.

HCP6 commented on meaningful worsening in f-SARA total score: *“I would think a worsening of 2 points over 2 years.”*

HCP2 commented on meaningful improvement in f-SARA total score: *“I think 1 point [change] will be meaningful, but 2 points change probably much more so, with the total score.”*

#### Meaningful Stability in f-SARA Scores

Stability in f-SARA score for individuals with SCA was considered meaningful by all HCPs, though meaningful time frames varied depending on individual patient disease courses. Stability across a 1- to 2-year time frame was regarded as clinically meaningful by most HCPs (*n* = 6/8). However, 1 HCP indicated that the time frame for meaningful stability should be considered in the context of f-SARA performance versus disease natural history. Another HCP considered that stability over a 1-year period may not be meaningful in a disease with slow progression, and the meaningfulness of differences between patients studied in a clinical trial should be used as reference. Considering the definition of stability, most HCPs (*n* = 6/7; 1 HCP was not asked) reported that a 0-point change on the f-SARA total score would indicate disease stability for individuals with SCA. However, 1 HCP reported that a 1-point fluctuation between annual visits can occur and did not agree that a 0-point change indicated true stability.

HCP1 commented on stability in the f-SARA score in individuals with SCA: *“If my exam shows exactly the same scoring, level 2 ability for walking from visit to visit over the course of a year, for instance, I would say that’s meaningful,”*

HCPs 1–5 (*n* = 5) reported that no change in f-SARA individual item scores over a 1- to 2-year time frame would be regarded as clinically meaningful. However, HCPs 6–8 (*n* = 3) reported that no changes in individual item scores had varying importance, which was dependent on the specific item, patient baseline level of impairment, and deviation from the natural history of SCA.

HCP6 commented on the meaningfulness of no change in the f-SARA score: *“I would consider no change a meaningful outcome if and only if via the natural history they would otherwise have worsened in this time frame.”*

HCP7 commented on the meaningfulness of no change in the f-SARA score: *“Of course, 1 year is enough [for no change to be meaningful]. But if you extend your observation period, the stability may be also more and more meaningful, of course.”*

### Capturing Meaningful Change and Stability Using the f-SARA: Perspectives from Individuals with SCA

Individuals with SCA were asked to describe what would constitute meaningful improvement and worsening related to each of the 4 f-SARA items based on their current level of disease severity (Supplementary Table 8).

#### Gait

Six of 7 individuals reported that maintaining their walking ability for a period of 1 year would be meaningful. Individuals who considered themselves as having mild difficulties focused on the ability to walk without stumbling or falling when describing meaningful change (*n* = 3/3). Those who considered themselves as having moderate difficulties or those who used walking aids, focused on switching to no aid use or less complex walking aids (*n* = 3/4). These descriptions of improvements are consistent with 0–1-point changes on the f-SARA Gait item. Most individuals with SCA (*n* = 4/5) defined meaningful worsening as the loss of independence, which would limit conduct of ADLs.

Individual 5 commented on meaningful improvement in the f-SARA Gait item: *“If I had stability and stay where I was, I’d be happy”.*

Individual 4 commented on meaningful worsening in the f-SARA Gait item: *“It would probably mean that I would have to lose the ability to live independently.”*

#### Stance

All individuals with SCA (*n* = 7) reported that no worsening in Stance symptoms for 1 year would be meaningful. Individuals generally reported that meaningful improvement would allow them to stand freely, without assistance or falling (*n* = 7/7). Most individuals (*n* = 6/7) considered meaningful worsening to be the loss of the ability to stand and the need for a wheelchair.

Individual 1 commented on whether stabilization of current ability in the f-SARA Stance item over a 1-year period is meaningful: *“Yeah, because it goes along with standing, walking, and everything else.”*

#### Sitting

All 7 individuals with SCA agreed that maintaining their current sitting ability for 1 year would be meaningful to them. Meaningful improvement was difficult to ascertain because most individuals did not have difficulty sitting. However, the ability to stand from a sitting position or to sit from a standing position without falling into a chair or without assistance was seen as a meaningful improvement for 3 individuals (*n* = 3/7). Two individuals with SCA3 (*n* = 2/7) considered meaningful improvement as maintaining their current ability to sit. Individuals with SCA described meaningful worsening as the loss of ability to participate in leisure activities (*n* = 2/7) and the need for assistance or support while sitting by (*n* = 2/7).

Individual 4 commented on meaningful worsening in the f-SARA Sitting item: *“Well, it would mean that I couldn’t watch TV, or sit on my trike, or ride the recumbent in the gym without needing support.”*

#### Speech

Five of 7 individuals with SCA stated that stability in their speech over 1 year would be meaningful. Overall, for those who considered themselves to have mild (*n* = 3/7) or moderate (*n* = 3/7) speech impairment, meaningful improvement was reported as being understood by others, not having to repeat oneself, speaking faster, articulating clearly, and requiring less energy during conversations. These descriptions of improvements are consistent with 0–1-point changes on the f-SARA Speech item. Five individuals with SCA (*n* = 5/7) described meaningful worsening as slurring words, having to repeat oneself more often, and not being understood by other people.

Individual 2 commented on meaningful worsening in the f-SARA Speech item: *“It would be if I was significantly slurring more, and people were asking me – if they wanted me to repeat myself again.”*

Individual 4 commented on meaningful worsening in the f-SARA Speech item: *“One, I’d have to stop working. And then 2, probably more important, my kids would probably not want to talk to me. And then who talks to me?”*

#### f-SARA Cognitive Debriefing

A full cognitive debriefing of all the components of the f-SARA was conducted with the HCPs (those previously exposed to the f-SARA [*n* = 5] and those newly exposed to the f-SARA [*n* = 3]) to confirm the content validity of the f-SARA. For each item (Gait, Stance, Sitting, and Speech), HCPs were asked about the f-SARA instructions (e.g., general ease of understanding and their interpretation of instructions), the f-SARA items (e.g., their interpretation and clarity of the items), and the f-SARA item response options (e.g., their interpretation and clarity of each item’s response options).

Nearly all HCPs understood the general instructions for each item and found the instructions easy to follow, with a couple of exceptions noted (e.g., 1 HCP found the Sitting item instructions difficult to follow, and 1 did not find the general instructions on the Gait and Speech items easy to follow). All HCPs correctly interpreted each item, their response definitions, and scoring instructions (Table 4 and Supplementary Table 10). The clarity of the response definitions was deemed good by most HCPs. The choice of response section on the 5-point ordinal scale was also described as easy by all HCPs for the Gait and Stance items; however, it was indicated as potentially difficult to clinically distinguish between certain response options for the Sitting and Speech items. In addition, some HCPs (all with previous exposure to the f-SARA [*n* = 5]) commented that the scoring may not be sensitive enough to capture small but meaningful changes in patient function.

**Table 4:** HCP cognitive debriefing of the items included on the f-SARA

Aspect of debriefing	f-SARA previously-exposed HCPs Agreement, *n*/*N* (%) (*N* = 5*)				f-SARA newly-exposed HCPs Agreement, *n*/*N* (%) (*N* = 3*)			
	Gait	Stance	Sitting	Speech	Gait	Stance	Sitting	Speech
**Item debriefing**								
Correct understanding of general instructions	5/5 (100.0)	5/5 (100.0)	5/5 (100.0)	5/5 (100.0)	3/3 (100.0)	3/3 (100.0)	2/3 (66.6)^a^	3/3 (100.0)
Ease of general instructions	3/4 (75.0)^a^	5/5 (100.0)	5/5 (100.0)	3/4 (75.0)^a^	3/3 (100.0)	3/3 (100.0)	3/3 (100.0)	3/3 (100.0)
Correct understanding of scoring instructions	5/5 (100.0)	5/5 (100.0)	5/5 (100.0)	5/5 (100.0)	3/3 (100.0)	3/3 (100.0)	2/2 (100.0)^b^	2/2 (100.0)^b^
Correct interpretation of item	5/5 (100.0)	4/4 (100.0)^b^	5/5 (100.0)	4/4 (100.0)^b^	1/1 (100.0)^b^	1/1 (100.0)^b^	2/2 (100.0)^b^	3/3 (100.0)

*In cases where the participant was not asked or did not answer the question, this is reflected in the denominator

^a^One HCP found the general instructions of the item difficult to follow and 1 HCP did not answer the question

^b^In 1 interview the question was not asked

Abbreviations: HCP healthcare professional, f-SARA modified functional Scale for the Assessment and Rating of Ataxia

HCP3 commented on f-SARA response options: *“A 0–4 scale is easier than the SARA, for sure. It means each question has the same weight, which in that regard makes it an improvement over the SARA.”*

HCP5 commented on f-SARA response options: *“The problem with it [the f-SARA] is that it is probably not appropriate for monitoring just very small, fine quantitative changes in a patient’s clinical function.”*

HCP8 commented on f-SARA response options: *“I think for example, in the Gait, the original SARA contains 8 items, which is perhaps too granular. So, reducing the number of items to score makes sense. And these items here do reflect the correspondence, perhaps more between a score and a functional milestone with, for example, needing support. So, the Gait item for me as a concept makes sense, reducing granularity, matching functional milestones.”*

## Discussion

The development and validation of a well-defined SCA-specific COA that reliably measures meaningful changes in the symptoms and daily functioning of individuals with SCA is important for the measurement of potential treatment benefits in the clinical trial setting. This study provides a comprehensive overview of the complexity and heterogeneity of the impact of symptoms in a sample of patients with SCA1 and 3 from the perspectives of the individuals with SCA and the HCPs who treat them. The findings support the content validity and clinical meaningfulness of the f-SARA for use by HCPs who treat individuals with SCA in a clinical trial setting.

Among the f-SARA concepts evaluated during the interviews, 3 of 4 concepts were reported to be relevant for SCA by all 7 individuals with SCA and all 8 HCPs. These 3 concepts were Gait, Stance, and Speech. Sitting was considered relevant by approximately half of the individuals with SCA but was reported as less important than other items in early disease. These results are consistent with data previously reported from surveys with individuals with SCA and their caregivers, who identified the f-SARA concepts that are most important to them: Gait (97.9–98.7%), Stance (73.4–79.3%), Speech (65.5–73.4%), and Sitting (6.9–8.9%) [40, 41]. The descriptions of mild, moderate, and severe SCA provided by HCPs and individuals with SCA were similar to the rating options for each f-SARA item, particularly those assessing Gait and Speech, suggesting that the items included on the f-SARA adequately reflect the temporal progression of SCA. All HCPs understood the general instructions, severity definitions, and rating/scoring instructions for the f-SARA. They agreed that the f-SARA was easy to administer, and that the items were clear and easy to score; however, there were some suggestions for improvement on the Sitting and Speech items.

Overall, HCPs and individuals with SCA reported that the f-SARA had the potential to detect clinically meaningful changes in symptoms of SCA, including stabilization. While individuals with SCA were not asked to provide a numerical value that would constitute a meaningful change in f-SARA score, most indicated that maintaining their current abilities for Gait and Speech for 1 year would be meaningful. There was disagreement between HCPs on what would represent meaningful change. HCPs with previous f-SARA exposure considered that a 1- to 2-point change on the f-SARA total score and a 1-point change on the item score was meaningful. Conversely, the f-SARA newly-exposed HCPs frequently referenced the need for natural history data to accurately define what constitutes meaningful change on both the total and item level f-SARA scores and had difficulty providing numerical values. However, whether previous exposure to the f-SARA or familiarity with natural history studies drove the differences between HCP cohorts remains unknown. Nonetheless, all HCPs, regardless of previous f-SARA exposure, agreed that f-SARA total score changes of 1–2 points would be considered meaningful for individuals with SCA.

While the Gait, Stance, and Speech concepts assessed in the f-SARA were determined to be relevant by HCPs and individuals with SCA, the Sitting concept was considered less relevant to SCA disease progression. Of those individuals with SCA participating in the interviews, most presented with mild-to-moderate SCA and had retained their ability to sit unsupported, which may have influenced their views on the relevance of the Sitting concept. Despite this, when considering the range of ADLs important to individuals with SCA, sitting may be a core ability that promotes retention of some independence. Similar to the individuals with SCA, some HCPs indicated that Sitting was less relevant across the spectrum of disease than other symptoms, particularly as stability in the sitting position is not impacted until later stage disease [45]. Interestingly, clinical studies have demonstrated that sitting abilities differ between SCA types, and individuals with SCA2 show significantly greater difficulties with sitting than those with other types of SCA [46]. While the concept of Sitting on the f-SARA was considered less relevant for individuals with SCA1 and 3 included in this study, it may be more applicable to those with other genotypes, particularly SCA2. Additional concepts such as manual dexterity (highlighted as particularly important by HCPs), vision impairments, cognition, mood, and work activities, which were relevant to individuals with SCA in this study, are not included on the f-SARA. The assessment of manual dexterity is included in the original SARA and reflects the interest to assess this concept in both clinical practice and trial settings [19, 47]. In addition, the Patient-Reported Outcome Measures (PROM)-Ataxia [40] and Brief Ataxia Rating Scale (BARS) measures [48] address more concepts than the f-SARA and may also be considered for evaluation of SCA disease progression. Further studies evaluating both the SARA and the f-SARA in tandem may provide insights into the relevance and validity of the instruments for disease progression. Despite this, the current study has identified the potential benefits of the f-SARA for evaluating disease progression in individuals with SCA over the course of a 12-month clinical trial, and for the assessment of therapies that might alter disease progression.

Natural history data are now available for the f-SARA, reported as a 1-point change over 1.5 years in individuals with early-stage SCA1, 2, 3, and 6 [49]. Further, Moulaire et al. [49] report that use of the f-SARA to detect clinically meaningful change in a 12-month interventional trial is a valid approach provided studies use larger sample sizes compared with those using the original SARA. A sample size of approximately 280 individuals with varying SCA subtypes (SCA1, 2, 3, and 6) was regarded as appropriate for a trial using the f-SARA, because this would account for the reduced sensitivity associated with the inclusion of only 4 items on the instrument, rather than the original 8 items in the SARA [49].

The development of a valid COA for use in the clinical trial setting that can reliably detect improvement of SCA symptoms caused by therapeutic intervention is of particular importance as the ataxia field moves towards phase 3 studies of multiple therapies [50]. There has been much debate in the field on the usefulness and relevance of the original SARA as the primary outcome measure in interventional trials. Indeed, a study investigating the clinical meaningfulness of the SARA from the perspective of individuals with mild-to-moderate SCA3 found that 25% of the total SARA score was overestimated by HCPs and did not reflect clinically meaningful impairments for individuals (with notable exception of the Gait item) [51]. Additionally, the Maas and van de Warrenburg study [51] indicated that modified versions of the SARA, which include reappraisals of scoring weights at the item level, are likely required to identify meaningful treatment effects [51]. While the f-SARA satisfies these criteria, further studies investigating whether the item 0–4 ordinal response scale detects treatment-dependent small but clinically meaningful changes are warranted.

Our findings confirm the content validity of the f-SARA and provide new evidence to support its use for evaluation of disease severity and progression in individuals with SCA in the clinical trial setting. Further studies are required to determine the sensitivity of the f-SARA to detect treatment effects, and to establish how the f-SARA may be used in conjunction with other COA instruments (i.e., those that measure manual dexterity or ADLs) to optimize future study design and data collection. We note the recommendation from some HCPs that the clinical relevance of the f-SARA could be refined and improved by including manual dexterity items such as finger–nose and finger chase. We emphasize, however, that this essentially reverts to the original SARA scale and does not incorporate insights from the analysis of US natural history data and the troriluzole phase 2 study, which suggest that appendicular items are less sensitive to change and/or are more variable over 1 year. Furthermore, the challenge with use of the original SARA instrument in a clinical trial enriched to capture changes in axial items is the absence of dynamics in appendicular scores in this population [52, 53].

Limitations of this study include the small sample size, convenience sampling, and limited inclusion of individuals with varying SCA subtypes. Sample bias may have been introduced because all individuals with SCA1 and 3 in the study were from the United States, and some were self-referred from a patient advocacy organization. The small sample size prevented data saturation being reached for some concepts in the interviews alone with individuals with SCA. Despite this, most concepts reached a form of data saturation through consideration of the qualitative patient survey data [40, 41]. Additionally, when the f-SARA was developed and the corresponding content validity interviews were designed, SCA3 was considered to be one of the most common genotypes of SCA in the United States and globally. We note that SCA27b may account for a substantial proportion of previously unexplained late-onset dominant and sporadic cerebellar ataxias [4, 5, 8, 9], and the relevance of the f-SARA in patients with this genotype has yet to be assessed. Most individuals (*n* = 6/7) in the current study had mild-to-moderate SCA3, which may limit the generalizability of the results to individuals with other SCA subtypes, particularly those with non-CAG repeat SCA subtypes. In addition, individuals with mild-to-moderate SCA3 may not have experienced the full spectrum of symptoms associated with SCA (i.e., those symptoms that manifest in later disease stages such as substantial difficulties with sitting), which could also have introduced bias during the concept elicitation phase. Further, while the interviews conducted with HCPs were designed to assess the broad spectrum of SCA, HCPs may have unconsciously provided responses related to SCA subtypes 1, 2, and 3 because of greater exposure to individuals with these more common subtypes.

## Conclusions

The f-SARA was developed based on recommendations from a regulatory body to capture unequivocal and compelling changes that might be functionally meaningful and reflect treatment effect in individuals with SCA. Our findings reveal that the f-SARA was well understood by HCPs and perceived to be relatively easy to implement. HCPs and individuals with SCA1 and 3 reported that the Gait, Stance, and Speech concepts included in f-SARA would detect clinically meaningful changes in SCA symptoms. Assessment of the concept of Sitting in early disease requires further consideration.

## Electronic Supplementary Material

Below is the link to the electronic supplementary material.

Supplementary Material 1

## Data Availability

The datasets generated during and analyzed during the current study are available from the corresponding author on reasonable request.
