# Supplementary material for: Content Validity of the Modified Functional Scale for the Assessment and Rating of Ataxia (f-SARA) Instrument in Spinocerebellar Ataxia
Source: Cerebellum. 2024 May 7;23(5):2012–27. doi: 10.1007/s12311-024-01700-2 (PMC11489265; doi:10.1007/s12311-024-01700-2)
Supplement: Supplementary file 1 — Supplementary Material 1 [file 12311_2024_1700_MOESM1_ESM.pdf]

Supplementary Material

Supplementary Table 1 HCP eligibility criteria for participation in qualitative interviews

	**f-SARA previously exposed**	**f-SARA newly exposed**
Age	≥ 18 years	≥ 18 years
Training	HCP with formal training who interacts with ≥ 1 individual with SCA at least twice per year on a professional basis	HCP with formal training who interacts with ≥ 1 individual with SCA at least twice per year on a professional basis
Language	Fluent in English to complete the consent process and interview	Fluent in English to complete the consent process; interview may be conducted in another language
Informed consent	Willing to provide informed consent	Willing to provide informed consent
Access	Access to computer video conference equipment	Access to computer video conference equipment
Time	Willing to participate in a 90–120-minute interview	Willing to participate in a 120–195-minute interview
f-SARA exposure	Must have taken part in the BHV-4157-206 phase 3 troriluzole efficacy trial and used the f-SARA instrument	No prior exposure to the f-SARA instrument in a clinical setting

Abbreviations: f-SARA modified functional Scale for the Assessment and Rating of Ataxia, HCP healthcare professional, SCA spinocerebellar ataxia

Supplementary Table 2 Eligibility criteria for individuals with SCA

Inclusion criteria	Exclusion criteria
Male and female participants aged 18–75 years	Diagnosis of any medical condition that may contribute significantly to the individuals' symptoms of ataxia or that may confound assessment of ataxia symptoms
Confirmed diagnosis (via genetic testing) of SCA1, SCA2, SCA3, SCA6, SCA7, SCA8, or SCA10	Individuals who have taken participated in the BHV-4157-206 phase 3 troriluzole efficacy trial
Minimum of 6 years of education and fluent in English language	Individuals suspected of fraudulent activity as concluded by the assessor (applicable to those recruited via patient advocacy groups and recruitment agencies only)
Adequate hearing, vision, and language skills to provide informed consent and participate in an interview	
Willing to provide written informed consent for participation in the study	
Access to video conference equipment	
Willing to participate in a 90-minute interview	

Abbreviations: f-SARA modified functional Scale for the Assessment and Rating of Ataxia, SCA spinocerebellar ataxia

Supplementary Table 3 Sample of the discussion guide for interviews with individuals with SCA

**Part 1: Concept elicitation**
General experience
Could you please walk me though all signs and symptoms you experience due to SCA day to day?
Which of these symptoms is most bothersome to you and why?
Please could you tell me about the impact(s) these symptoms have on your everyday life?<br>• Which of your daily activities are impacted by SCA?<br>• Which aspects of your everyday life are most affected by SCA?
What stands out to you as being particularly important or meaningful related to these issues?
<u>Instruction to interviewer</u>: After the individual has finished spontaneously describing symptoms and impacts and identified what they find particularly bothersome, important and/or meaningful, follow up with the probing questions for any symptoms or impacts that have not been spontaneously mentioned
**Example probes**
**Gait and walking**
Some individuals with SCA may also have difficulties with their gait and walking such as:<br>• Difficulties walking (e.g., abnormal gait, needing assistance to walk, unable to walk even supported)<br>• Requiring walking aids<br>• Trouble climbing the stairs<br>• Trouble turning around<br>• Inability to walk more than a few feet without stopping<br>Have you experienced any of these difficulties due to SCA? If so, which ones? Which are most important or meaningful to you? Which are the most bothersome?
Are there any other problems with gait or walking that have not been listed but are relevant to you?
Please could you tell me about the impact(s) your difficulties with gait and walking have on your everyday life?
**Falls**
Some individuals with SCA may experience falls<br>• Have you experienced any falling?
If patient says yes to experiencing falling, probe with the following questions
What is the frequency you experience falls (e.g., weekly, monthly, less)?
Please could you tell me about the impact(s) your difficulties with falls have on your everyday life? How has this affected your day-to-day activities?
**Stance and balance**
Some individuals with SCA may also have difficulties with their stance such as:<br>• Trouble standing (e.g., unable to stand in a natural position for a short period of time without assistance, able to stand for a short period of time but with assistance)<br>• Trouble keeping their balance<br>Have you experienced any difficulties with stance or balance due to SCA? If so, which ones? Which are most important or meaningful to you? Which are the most bothersome?
Are there any other problems with stance or balance that have not been listed but are relevant to you?
Please could you tell me about the impact(s) your difficulties with stance and balance have on your everyday life?
**Sitting**
Some individuals with SCA may also have difficulties with sitting such as:<br>• Difficulties sitting (e.g., an intermittent or constant sway, unable to sit without support)<br>• Requiring back support to sit<br>Have you experienced any difficulties with sitting due to SCA? If so, which ones? Which are most important or meaningful to you? Which are the most bothersome?
Are there any other problems with sitting that have not been listed but are relevant to you?
Please could you tell me about the impact(s) your difficulties with sitting have on your everyday life?
**Speech**
Some individuals with SCA may also have difficulties with their speech such as:<br>• Words may be occasionally difficult to understand

- Individual may be asked to repeat statements and frequency of this depends on the severity of the speech impairment
- Feeling frustrated when people don't understand what they are saying

Have you experienced any of these difficulties due to SCA? If so, which ones? Which are most important or meaningful to you? Which are the most bothersome?

Are there any other problems with speech that have not been listed but are relevant to you?

Please could you tell me about the impact(s) your difficulties with speech have on your everyday life?

Swallowing

Some individuals with SCA may also have difficulties swallowing such as:

- Choking (frequency depends on severity of swallowing impairment)
- Requires modified food to swallow more easily
- Avoid certain foods
- Coughing when eating

Have you experienced any of these difficulties due to SCA? If so, which ones? Which are most important or meaningful to you? Which are the most bothersome?

Are there any other problems with swallowing that have not been listed but are relevant to you?

Please could you tell me about the impact(s) your difficulties with swallowing have on your everyday life?

Personal care and other day-to-day activities

Some individuals with SCA may also need help with personal hygiene, dressing and other day-to-day activities such as:

- Needing assistance with personal hygiene (e.g., showering, brushing teeth, using the toilet)
- Issues with bladder function (e.g., urinary hesitance, urgency or retention, urinary incontinence)
- Unable to do their usual activities (e.g., hobbies, work)
- Needing help to do usual activities (e.g., hobbies, work)
- Difficulties dressing (e.g., occasional assistance with buttoning, getting arms in sleeves, may need to modify activity in some way as a result of this).
- Requiring assistance for cutting food and handling utensils (e.g., slower and sometimes clumsier when cutting foods, feeding speed may decrease)

Have you experienced any difficulties with personal hygiene, dressing, and other day-to-day activities due to SCA? If so, which ones? Which are most important or meaningful to you? Which are the most bothersome?

Are there any other problems with personal hygiene, dressing and other-day-to-day activities that have not been listed but are relevant to you?

Please could you tell me about the impact(s) the following difficulties have on your everyday life:

- Personal hygiene
- Dressing
- Cutting food and handling utensils
- Other day-to-day activities

Energy levels

Some individuals with SCA may also have difficulties with energy for instance:

- Feeling tired
- Lacking energy
- Feeling fatigued
- Feeling frustrated by being too tired to do the things they want to do

Have you experienced any difficulties with energy or fatigue due to SCA? If so, which ones? Which are most important or meaningful to you? Which are the most bothersome?

Are there any other problems with energy levels that have not been listed but are relevant to you?

Please could you tell me about the impact(s) your difficulties with energy levels have on your everyday life?

Part 2: Understanding meaningful change and appropriateness of symptoms and impacts in the context of SCA

On a scale of 0 to 4, where 0 is not at all relevant, 1 is a little relevant, 2 is somewhat relevant, 3 is very relevant and 4 is extremely relevant – where would you place the following symptoms in the context of your living with SCA?

- Difficulties with gait and walking
- Difficulties climbing the stairs

• Falls/sometimes falling down<br>• Difficulties turning around<br>• Difficulties standing<br>• Difficulties with balance<br>• Difficulties sitting<br>• Difficulties with speech<br>• Difficulties swallowing<br>• Coughing when eating<br>• Difficulties cutting food and handling utensils<br>• Difficulties dressing<br>• Personal hygiene<br>• Bladder function<br>• Fatigue/tiredness/lack of energy<br>• Inability to do usual activities / Needing help to do daily activities<br>• Frustration of being too tired to do the things I want to do<br>• Frustration when people don't understand what I am saying
Next, I would like you to pick 3 symptoms or disease impacts that are most important to you. Please rank them in order of importance: 1, the most important; 2, the second most important; and 3, the third most important. Please explain why each of these symptoms is important to you
Note to interviewer: If the patient choses an answer to that is not in the f-SARA (i.e., trouble climbing the stairs, falls, difficulties with balance, difficulties swallowing, coughing when eating, difficulties cutting food and handling utensils, difficulties dressing, issues with personal hygiene, bladder function, tiredness or fatigue, frustration due to tiredness, help doing usual activities or frustration with speech) – ask the following 5 questions:
What would you consider to be mild, moderate, severe and very severe difficulties with [*name symptom chosen by patient*]?
What level of difficulty [*name symptom chosen by patient*] do you think you are at? Please explain
What would be the smallest change(s) in difficulties [*name symptom chosen by patient*] that would be significant to *you*? For the worse and for the better?
What would a significant or meaningful worsening of [*name symptom chosen by patient*] mean to you?
Would a stabilization of your current level of [*name symptom chosen by patient*], over a year period, be a meaningful outcome to you?
Note to interviewer: The meaningfulness of change for all symptoms and impacts covered in the f-SARA will be discussed next. Before moving on to the next section ask the patient whether they would like a break. After the break or if the patient is happy to continue, move on to the next section
**Gait and walking**
What would you consider to be mild, moderate, severe and very severe difficulties with gait and walking?
What level of difficulty walking do you think you are at? Please explain
What would be the smallest change(s) in difficulties walking that would be significant to *you*? For the worse and for the better?
What would a significant or meaningful worsening of your walking ability mean to you?
Would a stabilization of your current level of walking, over a year period, be a meaningful outcome to you?
**Stance**
What would you consider to be mild, moderate, severe and very severe difficulties with standing?
What level of difficulty with standing do you think you are at? Please explain
What would be the smallest change(s) in difficulties standing that would be significant to *you*? For the worse and for the better?
What would a significant or meaningful worsening of your standing ability mean to you?
Would a stabilization of your current level of standing, over a year period, be a meaningful outcome to you?
**Sitting**
What would you consider to be mild, moderate, severe and very severe difficulties with speech?
What level of difficulty with speech do you think you are at? Please explain
What would be the smallest change(s) in difficulties with speech that would be significant to *you*? For the worse and for the better?

What would a significant or meaningful worsening of speech mean to you?
Would a stabilization of your current level of speech, over a year period, be a meaningful outcome to you?
**Speech**
What would you consider to be mild, moderate, severe and very severe difficulties with speech?
What level of difficulty with speech do you think you are at? Please explain
What would be the smallest change(s) in difficulties with speech that would be significant to *you*? For the worse and for the better?
What would a significant or meaningful worsening of speech mean to you?
Would a stabilization of your current level of speech, over a year period, be a meaningful outcome to you?

Supplementary Table 4 SCA symptoms, progression, and impact on daily function from the HCP perspective

HCP identity	Verbatim quotations regarding the impact of SCA on daily function
HCP1	*"One of the most common complaints that we get is falling, unexpected falls, and difficulty with mobility, standing, walking, whether it's outside the home or inside the home."*<br>*"The second most common complaint we get are changes in speech, where the person feels, for instance, by the end of the day, when they're tired, their speech may be slurred."*
HCP2	*"It affects all aspects of daily life: difficulty walking, difficulty in speaking, to be understood, and dexterity problems, and movement problems."*
HCP3	*"It affects all aspects of their daily life. The difficulty walking means that everything they do is more difficult. So, getting up to get a glass of water is more difficult. It impacts just all aspects of their function, really. It makes it much more difficult for them to even go to the bathroom."*
HCP4	*"It changes their independence, whether they can go out and get in the car by themselves and drive around, or that they have to have some equipment hauled with them where they go, and/or whether they need to have somebody else with them."*
HCP5	*"These patients are prone to fractures, and injuries, and falls. And then the other thing is, is that because it can be such a severe disease, they have tremendous loss of income. So, these are patients who are not able to be, generally speaking, gainfully employed. The other issue is that because most of them are autosomal dominantly related or inherited diseases, they also have to deal with the burden of their offspring, often having the same disease that they do. This is an unbelievably difficult emotional burden for them, causes a lot of guilt."*
HCP6	*"Gait is affected, balance and stance, fine motor skills, speech, swallowing, and sometimes eye movements. So, in terms of practical daily living, this means patients cannot walk straight, stumble when walking, sometimes stumble or sway when standing. They do have impaired fine motor skills, buttoning or handwriting, using knives and forks. And they have problems in swallowing, and they have problems in speaking, speaking unclearly, and sometimes as a correlate of the eye movements, they do have double vision."*
HCP7	*"The main burden for the patients is a lack of coordination. That means especially lack of balance, a gait disorder. These are the main points that can affect the daily living of the patients. Another main point is the speech disturbance. This may be very important for the patients. Because in the severe case of ataxia, you may have a speech that is not comprehensible or at limit of the comprehension."*
HCP8	*"For the severe disease stages, that is – you're losing your autonomy and independence, but also the early stages, people already, when they have mild symptoms, cannot keep on doing the work normally, cannot just ride a typical bicycle or drive a car. […] It's just not pure motor or mobility affecting disorder, but also some cognitive and affective problems might arise. So, it's a multi-domain progressive disease with increasing consequences."*

HCPs 1–5 had previous exposure to the f-SARA; HCPs 6–8 were newly exposed
Abbreviations: HCP healthcare professional, SCA spinocerebellar ataxia

Supplementary Table 5 Summary of sign, symptom, and impact concepts identified in interviews with individuals with SCA1 and 3

Concept	P1	P2	P3	P4	P5	P6	P7
**Gait and walking**							
Difficulties walking (including abnormal gait)[a]	S	S	S	S	S	S	S
Trouble climbing the stairs[a]	–	–	Pr	Pr	Pr	Pr	S
Sometimes required walking aids[a]	Pr	Pr	–	–	S	–	–
Trouble turning around[a]	–	–	–	Pr	Pr	Pr	–
Often requires walking aids[a]	–	–	–	S	–	S	–
Running	–	–	–	S	S	–	–
Stops when walking[a]	–	–	–	–	–	Pr	–
**Balance**							
General trouble keeping balance[a]	S	S	S	S	S	S	S
**Stance[b]**							
Trouble standing for long periods of time[a]	Pr	–	Pr	–	–	–	Pr
Needing standing support[a]	–	–	Pr	Pr	–	–	–
Difficulty getting out of chair/up from a sitting position[a]	–	–	–	–	Pr	–	S
Trouble standing due to pain[a]	–	–	S	–	–	–	–
Trouble standing upright[a]	–	S	–	–	–	–	–
Trouble standing on uneven/unstable surfaces[a]	–	–	–	–	Pr	–	–
Trouble standing[a]	–	–	–	–	–	S	–
Leg collapsing when standing[a]	–	–	–	–	–	S	–
**Sitting[b]**							
Difficulty sitting for long periods of time (e.g., due to pain, aches, or cramps; feeling stuck/sedentary)[a]	–	S	–	S	Pr	–	Pr
Requiring back support to sit[a]	–	–	–	Pr	–	–	Pr
Getting into a sitting position[a]	–	–	–	Pr	–	–	–
Difficulty sitting upright[a]	–	–	–	–	Pr	–	–
**Speech**							
Occasionally difficult to understand (e.g., slurred speech, speech production difficulties)[a]	S	Pr	S	Pr	Pr	S	S
Need to repeat statements and frequency[a]	–	–	Pr	–	Pr	–	–
Frustration with speech[a]	–	Pr	Pr	–	–	–	–
**Energy**							
Feeling tired or fatigued[a]	S	S	S	Pr	Pr	Pr	Pr
Lacking energy[a]	–	Pr	Pr	S	Pr	S	Pr
Frustration due to lack of energy[a]	–	–	Pr	–	Pr	–	–
Muscle weakness	–	–	–	–	Pr	–	–
**Emotion**							
Emotional dysfunction	Pr	S	S	Pr	S	Pr	S

Concept	P1	P2	P3	P4	P5	P6	P7
Frustration	–	–	S	–	Pr	–	–
Positive affect	–	–	S	–	Pr	–	–
Concern over future disability	–	–	–	S	–	–	S
**Usual activities**							
Difficulties with working[a]	S	S	S	S	Pr	Pr	S
Challenges with socializing or issues with social life	–	Pr	S	S	S	S	S
Difficulties dressing[a]	–	S	Pr	S	Pr	Pr	–
Difficulties with going for outings	S	–	Pr	–	–	S	Pr
Difficulties exercising	–	Pr	Pr	–	S	S	–
Difficulties driving	–	S	S	S	S	–	–
Difficulties with housework	S	–	Pr	–	S	Pr	–
Unable to do usual activities[a]	–	–	S	–	Pr	Pr	S
Difficulties travelling	–	Pr	–	–	S	Pr	–
Difficulties with activities involving bending	–	–	–	S	Pr	S	–
Difficulties with visual activities	–	S	–	–	S	S	–
Difficulties with family life	S	–	S	–	–	–	S
Difficulties cutting food and handling utensils[a]	–	–	Pr	–	–	Pr	–
Difficulties hiking	–	Pr	–	–	–	S	–
Difficulties with sports	–	S	–	–	–	S	–
Difficulties cooking	S	–	–	–	–	S	–
Difficulties drinking	–	–	–	–	Pr	–	S
Difficulties shopping	S	–	–	–	S	–	–
Difficulties sleeping	–	–	S	–	S	–	–
Difficulties with activities involving lifting	–	–	S	–	S	–	–
Difficulties biking	–	–	–	Pr	–	S	–
Difficulties eating	–	–	–	–	–	Pr	S
Needing help to do usual activities[a]	–	–	–	–	–	Pr	–
Difficulty playing an instrument	–	–	–	–	–	Pr	–
Difficulty learning	–	–	–	–	–	–	S
Difficulty dancing	–	–	–	–	–	–	S
**Falls**							
Sometimes falls[a]	S	–	S	S	S	–	–
Often falls[a]	–	–	–	–	–	S	S
**Swallowing**							
Choking[a]	S	–	Pr	–	S	Pr	S
Coughing when eating[a]	–	–	Pr	Pr	S	Pr	–
Requires modified food to swallow more easily[a]	–	–	–	–	S	Pr	Pr
Avoids certain foods[a]	–	–	–	–	–	–	–
**Vision**							

Concept	P1	P2	P3	P4	P5	P6	P7
Impaired focus/movement/sight in general	–	S	S	–	–	S	S
Double vision	–	S	S	–	–	S	–
**Neuropathy**							
General neuropathy	–	S	S	–	S	–	–
Muscle pain	–	S	S	–	P	–	–
Numbness/pins and needles	–	S	–	–	S	–	S
Muscle stiffness	–	–	S	–	–	–	–
Muscle cramps	–	–	–	–	–	–	Pr
**Cognition**							
Cognitive deficits	–	–	S	S	Pr	–	–
Memory deficits	–	–	S	–	Pr	–	–
**Bodily function**							
Issues with blader function[a]	–	–	Pr	S	Pr	Pr	–
Gastrointestinal issues	–	–	–	S	–	–	–
**Personal hygiene**							
Assistance with using the toilet[a]	–	–	S	Pr	Pr	Pr	–
Assistance with showering[a]	–	–	S	–	–	Pr	–
Assistance with brushing teeth[a]	–	–	–	–	–	Pr	–
**Dexterity**							
Fine motor control	–	–	Pr	–	–	S	–
Writing difficulties	–	–	–	–	S	S	–
**Muscle control**							
Muscle atrophy	–	–	S	–	–	–	–
Involuntary movements	–	S	–	–	–	–	–
**Sleep**							
Poor sleep quality	–	–	–	–	S	–	–
Movement restriction in bed	–	–	–	–	Pr	–	–
Need to nap/sleep often	–	–	–	–	–	Pr	–
**Sexual function**							
Loss of sex drive	–	–	Pr	–	–	–	–

– indicates the concept was not reported by the patient
Any concept that was mentioned upon probing (even if unrelated to the probed concept) was marked as probed
[a]Denotes concepts that were additionally probed
[b]Denotes concepts that were broken down during analysis to differentiate challenges with standing and sitting
Abbreviations: Pr probed, S spontaneous, SCA spinocerebellar ataxia

Supplementary Table 6 Verbatim quotations from participants with SCA1 and 3 on the most bothersome and important symptoms impacting daily function

Participant identity	Aspects of everyday life most impacted by SCA	Most bothersome symptoms	Most important or meaningful issues related to symptoms
P1	*"I guess doing daily activities. I mean, the fact that I still do them, but they're more difficult like cooking or like doing laundry, I do those things, but then it's a little more difficult."*	*"The fact that I have my gait, my balance issue, because with that, walking from point A to point B, and that's the most important thing that I need to do things around, to get out and do things."*	*"My balance, obviously, getting from point A to point B, being able to get around the house or wherever I want to go. And I hold onto things and just walk freely, that being more meaningful."*
P2	*"Well, being in pain. I can't sit and watch television. I can't because the pain is always worse at night."*	*"Neuropathy. Because I'm in pain 24 hours of the day."*	*"It's hard to be in pain all the time. I mean, you can correct your vision with glasses, but there really isn't a whole lot of medication that helps with the pain that doesn't put you to sleep or kind of make you really foggy. And if you need to work, you can't be foggy. So, that's pretty much the worst part."*
P3	*"It's kind of hard to say what's more affected than what's not affected, because it touches every single part of life. But I'd say just basic walking. I get up in the morning. It used to be that if I knew that I was gonna have a good balance day, if I wouldn't fall back into bed. But now it's more if I can just stand up and my feet don't hurt, then it's going to be a decent day with the neuropathy, with the pain."*	*"The neuropathy because it causes the most pain and the blepharospasms. I can do with the falls. I can do with the balance issues. I can work out and do something to combat that, but I can't combat neuropathy actively and I can't combat with blepharospasms actively."*	*"Working out and meditation. You have to make sure your mind is in the right place and you have to make sure your body's in the right place, or else you will digress every single day. And I've been able to, not get rid of the symptoms, but take something back or make them regress some by actually working out and training my mind and training my body every single day."*
P4	*"Working, I found that because it's harder to focus for more than 30 minutes without getting up and walking, that if you're working on a project, you've got to break it up."*	*"Using the rollator all the time isn't cool. It makes me feel like I'm really old. The doctor explained it as the ligaments. The natural walking of someone without ataxia has the natural reflexes. But when you have ataxia, your body doesn't recognize the steps as well. So, it collapses the need to prevent injury to the joint. That's how they explained it."*	*"I would like to be more social, but part of me comes back that it could be psychological, I would like to be more social, but I don't think – I feel as though people treat me different."*

Participant identity	Aspects of everyday life most impacted by SCA	Most bothersome symptoms	Most important or meaningful issues related to symptoms
P5	*"Exercise. Let's see. What else? Sleep. Those are 2 worst things. I can't run and I can't walk. I do exercise, but I have to do alternative exercise."*	*"Well, the neuropathy is the most bothersome because I cannot sleep."*	*"I do wish there was something, some kind of drug that I could take that would allow me to sleep. And then when I'm awake, I am not foggy for half a day or more."*
P6	*"I mean, in that you have to eat every day, I would say that is the most affected. I mean, it's hard to stand and to make your food to eat."*	*"My vision because it takes away my interaction with people. I mean, if you can't see faces, it's hard to interact with people."*	*"I feel like I'm not connecting with people because I can't see well enough to identify people."*
P7	*"The speech and walking."*	*"The speech. That's what bothers me the most."*	*"Knowing what's coming my way, of where I will be completely wheelchair-bound and not able to speak. Thinking that I may not have anybody to take care of me. And I won't be able to even speak to communicate."*

Abbreviations: P participant, SCA spinocerebellar ataxia

Supplementary Table 7 Summary of SCA1 and 3 symptoms considered most bothersome and important to participants by symptom domain

Symptom domain	Participants reporting domain *n* (%)	Concept	Participants reporting concept[a] *n* (%)	Most bothersome[b] (*n*/*N*)	Most important[c] (*n*/*N*)
Gait and walking	7 (100.0)	Difficulties walking	7 (100.0)	5/7	4/7
		Sometimes requires walking aids	3 (42.9)	2/3	3/3
		Often requires walking aids	2 (28.6)	2/2	0/2
		Trouble climbing the stairs	5 (71.4)	1/5	1/5
		Trouble turning around	3 (42.9)	1/3	0/3
		Stops when walking	1 (14.3)	1/1	1/1
Falls	6 (85.7)	Sometimes falls	4 (57.1)	N/A	N/A
		Often falls	2 (28.6)	N/A	N/A
Stance and balance[a]	7 (100.0)	Trouble standing	7 (100.0)	1/6	0/6
		Trouble keeping balance	7 (100.0)	3/6	2/7
Sitting[a]	4 (57.1)	Difficulties sitting (pain, trouble getting up from seated position, sudden cramps, and sitting on hard surfaces)	4 (57.1)	3/4	2/4
		Requiring back support to sit	2 (28.6)	0/4	1/2
Speech[a]	7 (100.0)	Occasionally difficult to understand	7 (100.0)	4/7	4/7
		Repeating statements and frequency	2 (28.6)	0/2	1/2
		Frustration with speech	2 (28.6)	0/2	0/2
Swallowing	6 (85.7)	Choking	5 (71.4)	3/5	2/5
		Coughing	4 (57.1)	0/4	3/4
		Requires modified food to swallow more easily	3 (42.9)	2/3	1/3
		Avoids certain foods	0 (0.0)	0	0
Personal care[a]	4 (57.1)	Assistance with showering	2 (28.6)	1/2	0/2
		Assistance with brushing teeth	1 (14.3)	1/1	0/1
		Assistance with using the toilet	4 (57.1)	1/4	0/4

**Symptom domain**	**Participants reporting domain** ***n* (%)**	**Concept**	**Participants reporting concept**[a] ***n* (%)**	**Most bothersome**[b] **(*n*/*N*)**	**Most important**[c] **(*n*/*N*)**
		Issues with bladder function	4 (57.1)	3/4	2/4
Usual activities[a]	7 (100.0)	Unable to do usual activities	4 (57.1)	1/4	0/4
		Needing help to do usual activities	1 (14.3)	1/1	0/1
		Difficulties dressing	5 (71.4)	1/5	0/5
		Difficulties cutting food and handling utensils	2 (28.6)	1/2	1/2
Energy	7 (100.0)	Feeling tired or fatigued	7 (100.0)	2/7	5/7
		Lacking energy	6 (85.7)	2/6	1/6
		Frustration due to lack of energy	2 (28.6)	0/2	0/2
Other symptoms	7 (100.0)	Social health	3 (42.9)	N/A	1/3
		Sleep issues	2 (28.6)	1/2	1/2
		Neuropathic pain	3 (42.9)	2/3	N/A
		Frustrated	1 (14.3)	1/1	N/A
		Not working	7 (100.0)	1/7	N/A
		Housework	4 (57.1)	1/4	N/A

[a]Total count of participants is $N = 6$ for the bothersome and important questions; participants either mentioned not experiencing any of the symptoms within the domain or had already discussed them spontaneously
[b]Total number of participants reporting the symptom as most bothersome within each domain
[c]Total number of participants reporting the symptom as most important within each domain
Abbreviations: N/A not applicable, SCA spinocerebellar ataxia

Supplementary Table 8 Verbatim quotations of f-SARA meaningful change from the perspective of participants with SCA1 and 3

f-SARA concept	Participant identity	Participant's current level of difficulty with concept	Meaningful improvement	Meaningful worsening	Is stabilization of current ability over a 1-year period meaningful?
Gait	P5	Moderate	*"If I had stability and stay where I was, I'd be happy."*	*"Well, I don't want to be in a wheelchair where I'm depending on somebody. Well, if I can't go someplace on my own, then I'm going to be depending on somebody to get me there. And I like my independence. I guess it would be independent movement."*	*"Yeah, that would be acceptable. I don't think that I'm going to be able to walk again. I understand my difficulties."*
Stance	P1	Moderate	*"Standing, maybe being able to stand without assistance? I don't know. I don't need that assistance. Standing freely."*	*"I mean, I won't be able to get up or stand. I mean, that would be horrible, because that would mean everything else went down. Because standing requires you to be able to walk, right?"*	*"Yeah, because it goes along with standing, walking, and everything else."*
	P6	Experiences all stages from mild to severe	*"The fatigue, standing without fatigue would be the lowest, the smallest thing that would be meaningful."*	*"Just the opposite, where I just fatigue constantly where I can't stand, it's too fatiguing."*	*"Yes. Goodness, yes."*
Sitting	P3	Mild	*"I'd be okay with just maintaining my current ability to sit because I really just want to sit right now."*	*"If I couldn't sit upright and hold my body still, that'd be pretty frustrating."*	*"Yes."*
	P5	Moderate	*"It'd be nice if I can go to the point where I can go anywhere, travel anywhere. But I'd accept stability and my condition doesn't get any worse."*	*"Well, I guess any noteworthy limitation of the distance I could travel while sitting would be unacceptable."*	*"Yes."*
Speech	P6	Mild	*"Being able to speak faster."*	*"Yeah, worsening would be when the first person I talk to says, 'Would you repeat yourself?' I*	*"Yeah, stabilization of this thing is good in all cases. I can't imagine... I mean, if I could*

				didn't hear. I couldn't understand you."	*stabilize, if I could guarantee for a year that I wouldn't wake up being worse off than I was the day before. It would be good."*

Abbreviations: f-SARA modified functional Scale for the Assessment and Rating of Ataxia, P participant, SCA spinocerebellar ataxia

Supplementary Table 9 Capturing meaningful change using the f-SARA

HCP identity	f-SARA item score change	f-SARA total score change	Verbatim quotations
**HCP definitions of meaningful improvement for SCA using the f-SARA**			
HCP1	1	1	*"I think a change certainly for Gait, Stance, and Sitting, a full point for any of these areas would obviously be clinically meaningful."*
HCP2	1	1–2	*"I think 1 point will be meaningful [with the total score], but 2 points change probably much more so."*
HCP3	1	1–3	*"It's not just 1 point necessarily, it's where that 1 point is. So, going from a 14 to 13 is probably not meaningful to anybody, but going from a 6 to a 5 might actually mean something to a patient."*
HCP4	1	2.2	*"I should say [a 2.2 change] on total – over whatever period of time, I think that would be a real change. Whereas a change of 1, it's not a very big change. It can be a close judgment call and it may not be real."*
HCP5	1	2–4	*"If you improved 1 point on each, which would be amazing, I would say a 2 to 4-point improvement. 4 would be extremely optimistic. One point, just 1 point for 1 or 2 of these would be meaningful."*
HCP6	1–2 (item dependent)	2	*"On the total score, it's an improvement of plus 2. I would be quite sure that this is meaningful."*
HCP7	1	1	*"If you have patients that in 1 year tend to progress of 2 points at SARA score, and after in the intervention group of patients, you observe an improvement of 1 point of SARA score, this is for me an endpoint of efficacy, a suggestion of effectiveness of those therapeutic approaches."*
HCP8	1–2 (item dependent)	1	*"So, delta one for the Gait going down. That is clear improvement, I would say. For the Stance, it depends a bit where you're from. But again, if you are able to change from in need of support to going down to no need of support, that is a relevant change. Then you go from score 1 to 0, the question is whether that is having any meaningful effect that the patient perceives, him or herself. Same as for Sitting. Same as for Speech."*
**HCP definitions of meaningful worsening for SCA using the f-SARA**			
HCP1	1	1	*"I think going from a scoring level to the next worsened scoring level or the next beneficial scoring level, as we've discussed, is something that's going to be easy for the examiner to rate and to be aware of."*
HCP2	1	1	*"I guess 1 point or something would be meaningful."*

HCP identity	f-SARA item score change	f-SARA total score change	Verbatim quotations
HCP3	1	1–3	*"I would say at least 1 or 2 points, but I think, again, it depends on where those 1 or 2 points are. If you're going from 13 to 15, I don't know how meaningful that is. It might be. But if you're going from 0 to 3, that's probably has huge meaning to patients."*
HCP4	1	2.2	*"I would say that somebody has truly altered, they've really lost ground, if they have a decline in their total f-SARA of 2.2."*
HCP5	1	2–4	*"One point on each of these items, so 2- to 4-point worsening."*
HCP6	1	1–2	*"Across the 4 item domains a total change of 2 points, then I would say this sums up to something which is of meaning for patients."*
HCP7	HCP7 did not believe this was quantifiable	1–2	*"I can expect that a patient with SCA1 may change 1 or 2 points in 48 weeks also with this form of modified SARA items."*
HCP8	1	1	*"Well, I think a score of 1 on the 16 could be clinically relevant."*
**HCP definitions of point changes associated with stability for SCA using the f-SARA with comments on whether stability is meaningful**			
HCP1	0	0	*"If somebody were taking a medication or were proposing to take a medication in the early or middle stages of a disease, and their scoring level for walking, for instance, stay stable for the course of a year, that would be a meaningful goal to reach with a medication."*
HCP2	0	0	*Question from interviewer: You mentioned that no change is considered stable for the total score. Would this be the same for the item level?"*<br>*HCP2 response: "Oh, yeah, exactly."*
HCP3	0	0	*"So, total score stability. I mean, I think they're both meaningful [item and total score]. They're meaningful in slightly different ways. I think they're both meaningful."*
HCP4	0	0	*"I think it would be meaningful for the total score and at the item level, but it's probably better at that total score because it covers more areas. In other words, stability, your gait doesn't change over time, we would expect your stance also not to change if you're stable. But if one changed and one didn't, then you've got some ambiguity about what exactly is happening with the patient. But the total score encompasses that because it's a total score. So, I think the total score is more reliable than an individual element."*
HCP5	0	0	*"Yes, [no change] over a significant amount of time [would be considered stable]."*
HCP6	0	0	*"Yes, no change [in item score] would be considered a stable response."*

<table>
<tr><th>HCP identity</th><th>f-SARA item score change</th><th>f-SARA total score change</th><th>Verbatim quotations</th></tr>
<tr><td>HCP7</td><td>0</td><td>Not asked</td><td>Question from interviewer: “So, no change in 48 weeks in the item score. Would that be meaningful?”
HCP7 response: “Yeah, I think so.”</td></tr>
<tr><td>HCP8</td><td>> 0</td><td>> 0</td><td>“It depends a bit on which SCA types. That might be too much detail for here, but we know there are different progression rates for some of these different SCA types. Some are very slow. And then 48 weeks is quite short. Some SCA types are a bit more aggressive, and then you would expect a change after a year or 48 weeks perhaps. But as a general reflection, stabilization does not necessarily reflect a meaningful endpoint to me.”</td></tr>
<tr><td colspan="4">HCP comments on timeframes for meaningful stability for SCA in f-SARA scoring</td></tr>
<tr><td>HCP1</td><td colspan="3">“If my exam shows exactly the same scoring, level 2, ability for walking from visit to visit over the course of a year, for instance, I would say that’s meaningful. Now, the patient themselves may have other things that are going to bring into the discussion, but on the basis of this rating scale, it would reflect stability.”</td></tr>
<tr><td>HCP2</td><td colspan="3">“If you say no change over 1 year or no change over 2 years, 3 years, it’s probably meaningful for disease-modifying therapy. But if it’s symptomatic therapy, no change, probably with a short period of time probably means it’s not meaningful.”</td></tr>
<tr><td>HCP3</td><td colspan="3">“I think no change over the course of – it depends on what time period. So, no change over the course of a year would be incredibly meaningful, absolutely.”</td></tr>
<tr><td>HCP4</td><td colspan="3">“I would say in the SCA, patients who are talking about 24 months with no change. I’d say that would probably be meaningful. We see within the 2-year span, virtually everybody loses ground.”</td></tr>
<tr><td>HCP5</td><td colspan="3">“So, no change over a year, in that context, it’s very meaningful.”</td></tr>
<tr><td>HCP6</td><td colspan="3">“I would consider this a meaningful outcome if, and only if, via the natural history they would otherwise have worsened in this timeframe and would have worsened by that delta.”</td></tr>
<tr><td>HCP7</td><td colspan="3">Question from interviewer: “So, no change in 48 weeks in the item score. Would that be meaningful?”
HCP7 response: “Yeah, I think so.”</td></tr>
<tr><td>HCP8</td><td colspan="3">“Depends on previous data on natural history to give an answer based on facts, not just intuition. But at least 2 years, I think I said the 1 year is short, many of these SCA types. So at least 2 years, and to that, I would like to refer more to the SARA or f-SARA total than to individual items.”</td></tr>
</table>

HCPs 1–5 had previous exposure to the f-SARA; HCPs 6–8 were newly exposed

Abbreviations: f-SARA modified functional Scale for the Assessment and Rating of Ataxia, HCP healthcare professional, SCA spinocerebellar ataxia

Supplementary Table 10 HCP cognitive debriefing of the response options included on the f-SARA

Aspect of debriefing	f-SARA previously-exposed HCPs Agreement, *n*/*N* (%) (*N* = 5*)				f-SARA newly-exposed HCPs Agreement, *n*/*N* (%) (*N* = 3*)			
	**Gait**	**Stance**	**Sitting**	**Speech**	**Gait**	**Stance**	**Sitting**	**Speech**
**Response options debriefing**								
Definition of normal (no impairment)								
Correct interpretation	5/5 (100.0)	5/5 (100.0)	5/5 (100.0)	5/5 (100.0)	3/3 (100.0)	3/3 (100.0)	2/2 (100.0)[a]	2/2 (100.0)[a]
Clarity	5/5 (100.0)	5/5 (100.0)	5/5 (100.0)	5/5 (100.0)	2/2 (100.0)[a]	3/3 (100.0)	3/3 (100.0)	2/2 (100.0)[a]
Definition of mild impairment								
Correct interpretation	5/5 (100.0)	5/5 (100.0)	5/5 (100.0)	5/5 (100.0)	3/3 (100.0)	2/2 (100.0)[a]	2/2 (100.0)[a]	2/2 (100.0)[a]
Clarity	4/5 (80.0)	4/5 (80.0)	4/4 (100.0)[a]	4/4 (100.0)[a]	1/3 (33.3)	2/3 (66.7)	3/3 (100.0)	2/2 (100.0)[a]
Definition of moderate impairment								
Correct interpretation	5/5 (100.0)	5/5 (100.0)	5/5 (100.0)	5/5 (100.0)	3/3 (100.0)	2/2 (100.0)[a]	2/2 (100.0)[a]	2/2 (100.0)[a]
Clarity	4/5 (80.0)	5/5 (100.0)	5/5 (100.0)	5/5 (100.0)	1/3 (33.3)	2/3 (66.7)	1/2 (50.0)[a]	2/2 (100.0)[a]
Definition of severe impairment								
Correct interpretation	5/5 (100.0)	5/5 (100.0)	5/5 (100.0)	5/5 (100.0)	3/3 (100.0)	2/2 (100.0)[a]	2/2 (100.0)[a]	2/2 (100.0)[a]
Clarity	5/5 (100.0)	5/5 (100.0)	5/5 (100.0)	5/5 (100.0)	3/3 (100.0)	2/2 (100.0)[a]	2/2 (100.0)[a]	2/2 (100.0)[a]
Definition of patient unable to walk/stand/sit/speak intelligibly								
Correct interpretation	5/5 (100.0)	5/5 (100.0)	5/5 (100.0)	5/5 (100.0)	3/3 (100.0)	2/2 (100.0)[a]	2/2 (100.0)[a]	2/2 (100.0)[a]

**Aspect of debriefing**	**f-SARA previously-exposed HCPs Agreement, *n*/*N* (%) (*N* = 5*)**				**f-SARA newly-exposed HCPs Agreement, *n*/*N* (%) (*N* = 3*)**			
	**Gait**	**Stance**	**Sitting**	**Speech**	**Gait**	**Stance**	**Sitting**	**Speech**
Clarity	5/5 (100.0)	5/5 (100.0)	5/5 (100.0)	5/5 (100.0)	3/3 (100.0)	2/2 (100.0)[a]	2/2 (100.0)[a]	2/2 (100.0)[a]
Selecting a response option								
Ease of selection response option	5/5 (100.0)	5/5 (100.0)	3/5 (60.0)	4/5 (80.0)	3/3 (100.0)	3/3 (100.0)	3/3 (100.0)	1/3 (33.3)

*In cases where the participant was not asked or did not answer the question, this is reflected in the denominator

[a]In 1 interview the question was not asked

Abbreviations: f-SARA modified functional Scale for the Assessment and Rating of Ataxia, HCP healthcare professional
